# Advancements in Genetic Marker Exploration for Livestock Vertebral Traits with a Focus on China

**DOI:** 10.3390/ani14040594

**Published:** 2024-02-11

**Authors:** Muhammad Zahoor Khan, Wenting Chen, Bingjian Huang, Xiaotong Liu, Xinrui Wang, Yihong Liu, Wenqiong Chai, Changfa Wang

**Affiliations:** Liaocheng Research Institute of Donkey High-Efficiency Breeding and Ecological Feeding, Liaocheng University, Liaocheng 522000, China

**Keywords:** livestock, vertebral traits, meat production, genetic markers, genomic selection

## Abstract

**Simple Summary:**

In the context of increasing global meat consumption, the meat production industry is facing significant challenges as it strives to enhance attributes related to livestock and meat quality. This is of paramount importance. Vertebral traits are among the factors influencing the overall body size and carcass weight of animals, with profound implications for both meat quality and quantity. This issue is particularly prominent in the livestock breeding sector, especially in China, where the augmentation of body size traits has become a central objective due to its consequential impact on carcass quality. Vertebral traits in livestock are complex, polygenic characteristics, and numerous genetic markers and pathways associated with vertebral development, size, and length have already been identified. This review article emphasizes the significant genes linked to vertebral traits in animals such as pigs, sheep, and donkeys based on the existing literature. It further underscores the importance of exploring deeper into molecular mechanisms to gain a more comprehensive understanding of the relationship between vertebral traits and genes, facilitating their effective utilization in successful breeding programs.

**Abstract:**

In livestock breeding, the number of vertebrae has gained significant attention due to its impact on carcass quality and quantity. Variations in vertebral traits have been observed across different animal species and breeds, with a strong correlation to growth and meat production. Furthermore, vertebral traits are classified as quantitative characteristics. Molecular marker techniques, such as marker-assisted selection (MAS), have emerged as efficient tools to identify genetic markers associated with vertebral traits. In the current review, we highlight some key potential genes and their polymorphisms that play pivotal roles in controlling vertebral traits (development, length, and number) in various livestock species, including pigs, donkeys, and sheep. Specific genetic variants within these genes have been linked to vertebral development, number, and length, offering valuable insights into the genetic mechanisms governing vertebral traits. This knowledge has significant implications for selective breeding strategies to enhance structural characteristics and meat quantity and quality in livestock, ultimately improving the efficiency and quality of the animal husbandry industry.

## 1. Introduction

The meat production industry encounters challenges in augmenting carcass and meat quality attributes, significantly influencing consumer preferences and demand amid the global upsurge in meat consumption [1,2]. Key attributes encompassing body morphology, skeletal architecture, vertebral characteristics, muscular development, tenderness, and adipose deposition in livestock species hold paramount significance in governing both meat quality and quantity [3,4,5,6,7,8,9].

In the domain of livestock breeding, the pursuit of augmenting traits related to body size has emerged as a central objective, particularly in China, due to its substantial influence on meat production and carcass traits [10,11,12]. Recent research underscores the economic significance of vertebral count in determining carcass length, weight, and body size traits [10,11,12,13]. Variations in body size have emerged within and between domestic animal species or breeds during livestock evolution [14]. Notably, the number of thoracolumbar vertebrae plays a crucial role in carcass characteristics, particularly carcass length [15]. Across different pig breeds [16], sheep breeds [17], donkeys [10,18], and yaks [19], variations in the number of thoracolumbar vertebrae have been observed. This trait has been considered a selection criterion in commercial animal breeding due to its strong correlations with growth and meat production, exhibiting high heritability (0.60–0.62) and a positive correlation with body length [15].

China has made significant advancements in livestock genetic improvement, particularly in pig breeding [20]. However, enhancing breeding efficiency and accuracy remains a challenge. Molecular marker techniques, such as marker-assisted selection (MAS), have emerged as a rapid and effective approach [21,22,23]. MAS relies on the linkage between phenotypic traits and molecular markers within the genome, enabling the swift and accurate identification of individuals with desired traits, thus improving breeding efficiency. Identifying candidate genes and their polymorphisms associated with vertebral traits holds scientific and economic significance, providing markers for genetic enhancement in livestock.

The influence of genetic factors and signaling pathways on multi-vertebrae traits in livestock is a complex and multifaceted area of research. Several studies have shed light on the genetic underpinnings of these traits, with a focus on genes such as *PLAG1*, *VRTN*, *PRKG2*, *MMP4*, *NR6A1*, *LTBP2*, *DCAF7*, *NCAPG-LCORL*, *ActRIIB*, and *TGFβ3*, as well as their associations with specific vertebral characteristics in various livestock species, especially pigs, donkeys, and sheep. Consistently, Yan et al. [10] and Liu Z et al. [18] emphasized the quantitative nature of these traits, indicating that they are influenced by multiple genes and intricate biological signaling pathways. This highlights the polygenic nature of multi-vertebrae traits, where numerous genetic factors interplay to determine the final outcome. The genes *PLAG1* and *NCAPG-LCORL*, originally known for their roles in human height, carcass weight, and body length, have emerged as key players in livestock vertebral traits, underscoring their pleiotropic effects [24]. Furthermore, specific mutations in genes like *ActRIIB* and *TGFβ3* have been linked to variations in vertebral number in sheep and pigs, respectively. Liu J et al. [25] identified a point mutation in intron 4 of the ActRIIB gene in Small-Tailed Han sheep associated with vertebral number variation. Similarly, Yue J et al. [26] reported that the mutation g.105179474 G  > A in the TGFβ3 gene was associated with rib and thoracolumbar vertebrae numbers in pigs, highlighting the genetic diversity that can underlie these traits. In Beijing Black pigs, Niu N et al. [27] identified a significant link between variant-g.19034 A  >  C of the *VRTN* gene and multiple thoracic vertebrae numbers, further exemplifying the genetic complexity of vertebral traits in sheep and pigs.

Donkeys are a comparatively less explored area in this context and have shown promising insights. Shi et al. [28] reported significant associations between specific genetic loci, *HOXC8* g.15179224C  >  T and g.15179674G  >  A, with lumbar vertebrae length and the number of lumbar vertebrae. This highlights the potential for genetic selection and breeding strategies to influence vertebral traits in donkeys, similar to other livestock species. Nevertheless, the existing body of literature to date has exclusively focused on the investigation of genetic markers associated with vertebral traits in donkeys, sheep, and pigs. However, it is noteworthy that comprehensive investigations into the association of genetic markers with vertebral traits in cattle and horses have been absent from the existing body of research. Drawing upon the aforementioned evidence, it becomes evident that variations in vertebral traits among livestock species (donkeys, sheep, and pigs) can significantly influence changes in body size, carcass length, and weight. Furthermore, it is essential to recognize that vertebral traits are inherently quantitative in nature, controlled by a complex interplay of multiple genes. Thus, the exploration and screening of genes associated with these vertebral traits hold substantial potential for enhancing meat production within the livestock sector, thereby making a notable contribution to the meat industry. Therefore, the primary objective of this review article is to elucidate the recent advancements in the study of genetic markers linked to vertebral traits in pigs, donkeys, and sheep. The purpose is to provide comprehensive insights that can serve as foundational references for future research endeavors within this domain.

## 2. Methodology and Criteria for Literature Search and Selection

In the course of this current review, our methodology for the selection of relevant literature was established with careful consideration. We focused our attention primarily on articles that had been published from the year 2015 to the present day. To broaden the scope of our discussion, we also took into account pertinent data from articles published after the year 2000. Our search strategy entailed the utilization of specific keywords to identify relevant articles. The keywords employed encompassed subjects pertaining to livestock, specifically pigs, sheep, and donkeys. Additionally, we sought articles encompassing genetic markers, vertebral traits, growth traits, and both the quality and quantity of meat. It is worth noting that we exercised selectivity in our inclusion criteria. Articles published in journals not indexed in the Science Citation Index (SCI), books, or book chapters, as well as those published in languages other than English, were excluded from our review.

## 3. Genetic Markers Associated with Number of Vertebrae in Pigs, Donkeys, and Sheep

### 3.1. Genetic Markers Associated with Number of Vertebrae in Pigs

Pig vertebral classification comprises five distinct segments: cervical, thoracic, lumbar, sacral, and caudal vertebrae. The precise count of cervical, sacral, and caudal vertebrae in pigs is consistent at seven, four, and five, respectively [15]. The key constituents of the vertebral column are the thoracic and lumbar vertebrae, exhibiting considerable variability in their numbers. In Western modern breeds, the thoracic vertebral number spans from 13 to 17, while the lumbar vertebral number ranges from five to seven [13,15]. Wild boars possess 19 thoracic-lumbar vertebrae, whereas Chinese indigenous breeds exhibit a total thoracic and lumbar vertebral count ranging from 19 to 20 [13,15]. Notably, Western commercial breeds such as Large White, Duroc, and Landrace feature a higher number of thoracic-lumbar vertebrae (*n* = 21 to 23) due to rigorous selective breeding [13,15]. In pigs, some causal or tightly linked genes affecting crucial vertebral traits and used in practical production have been reported in previous studies [29,30,31,32]. In practical pig production, the integration of genetic knowledge regarding vertebral traits allows producers to make informed breeding decisions. Selecting breeding stock based on favorable genetic markers can lead to more robust, efficient, and economically viable pig populations. In addition, vertebral malformations can lead to structural deformities, reduced growth rates, and increased susceptibility to injuries. This, in turn, contributes to the sustainability and competitiveness of the swine industry. The summary of genes associated with vertebral traits is provided in Table 1.

### 3.2. Genetic Markers Associated with Number of Vertebrae in Donkeys

**Figure 1:**
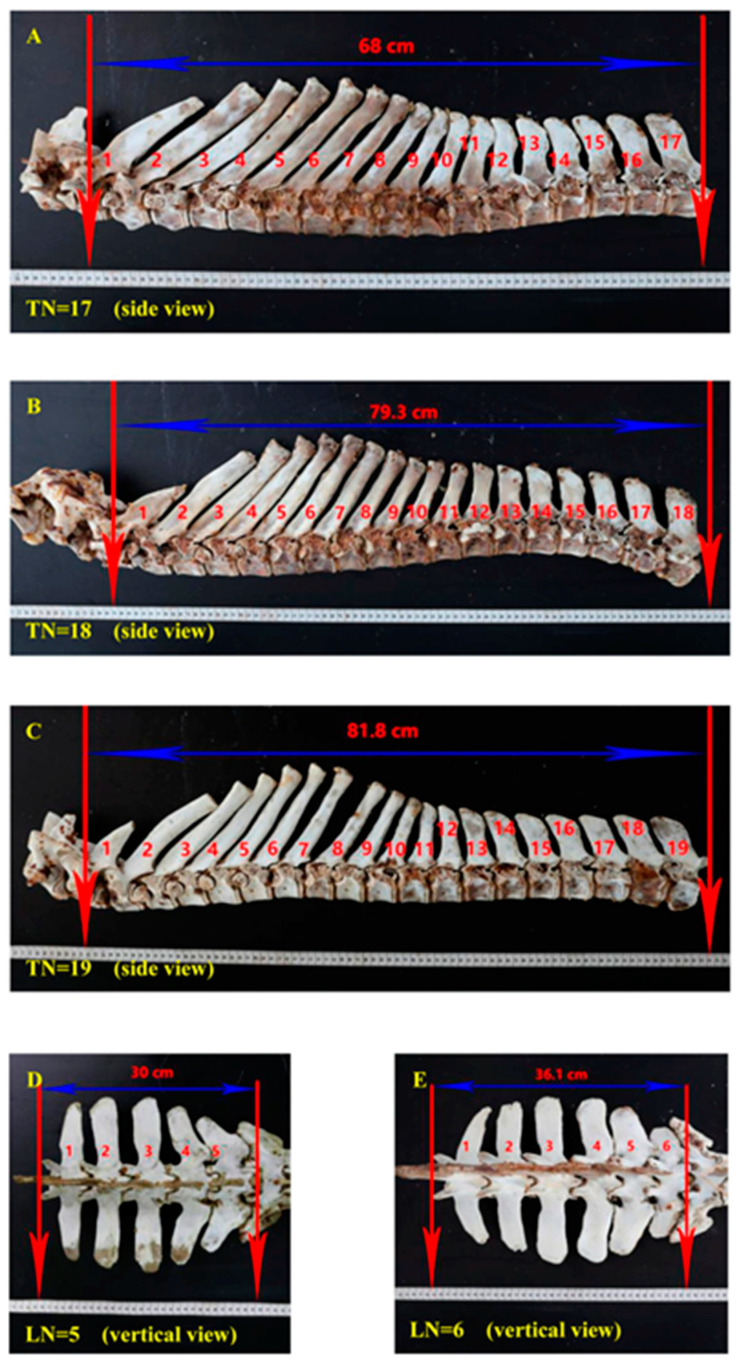
Association of number of vertebrae with body size traits [46]. (**A**) Side overview of 17 thoracic vertebrae; (**B**) Eighteen thoracic vertebrae side overview of seventeen; (**C**) Side overview of 19 thoracic vertebrae; (**D**) Five lumber vertebrae vertical overview; (**E**) Six lumber vertebrae vertical overview.

The study by Liu Z et al. [18], which involved a comprehensive survey of 455 donkeys, highlighted the presence of diverse configurations of thoracic and lumbar vertebrae in the Dezhou donkey population [18]. These configurations were identified as T18L5, T18L6, T17L6, T17L5, and T19L5. Notably, T18L5 was the most prevalent, accounting for 75.8% of the population. This finding suggests a certain level of variability in vertebral numbers among Dezhou donkeys. Moreover, research conducted by Liu Z et al. also established a correlation between the body size and weight of donkeys and the number of vertebrae (Figure 1) [18,45]. This correlation could potentially open up avenues for selective breeding and manipulation of vertebral numbers to enhance the structural characteristics of donkeys within the industry. However, it is important to note that the genetic mechanisms governing these traits require further investigation.

In the context of understanding the genetic basis of vertebral number determination in donkeys, recent studies have made significant contributions [12,18,45,46]. Specifically, the *PRKG2* gene has emerged as a key player associated with both the number and length of thoracic and lumbar vertebrae in donkeys [12]. Specific genetic variants within *PRKG2* were found to be significantly correlated with thoracic and lumbar vertebrae numbers as well as their length. This genetic insight provides valuable information for further research into the molecular mechanisms behind vertebral development.

It is worth noting that *PRKG2,* located on chromosome 3, consists of 18 exons and 17 introns in donkeys, as outlined by Wang C et al. [22]. Interestingly, *PRKG2* has also been implicated in dwarfism in various species, including American Angus cattle [47], humans [48,49], and dogs [50], underscoring its importance in skeletal development across different organisms. Additionally, its involvement in adipocyte and osteoblast differentiation in the human body, as reported by Yi et al., highlights the multifaceted nature of *PRKG2*’s functions [51]. In conclusion, the research on vertebral variations in Dezhou donkeys, coupled with the identification of the *PRKG2* gene and its associated genetic variants, provides a foundation for further exploration into the genetic mechanisms governing vertebral development. The summary of genes associated with vertebral traits in donkeys is provided in Table 2.

### 3.3. Genetic Markers Associated with Number of Vertebrae in Sheep

In the context of sheep anatomy, sheep typically possess a vertebral arrangement comprising seven cervical vertebrae (C), 13 thoracic vertebrae (T), six lumbar vertebrae (L), and four sacral vertebrae (S), totaling 30 vertebrae. Notably, mutations in the thoracolumbar region, such as T14L6 or T13L7, have been identified as the most prevalent [52]. These mutations are associated with multi-vertebrae sheep, which exhibit enhanced adaptability and meat production performance [52]. The cultivation of such multi-spine sheep carries substantial benefits for the economy, society, and ecology, making it a pivotal endeavor for improving the quality and efficiency of the animal husbandry industry.

In the case of Kazakh sheep, an indigenous breed in Western Xinjiang, China, there exists variability in the number of lumbar vertebrae. While most sheep conform to the standard configuration of 13 thoracic vertebrae and six lumbar vertebrae, denoted as T13L6, Kazakh sheep exhibit variations, specifically in T13L7 and T14L6. These variations correspondingly lead to an increase in carcass length by 2.22 cm and 2.93 cm compared to the typical T13L6 Kazakh sheep. Moreover, there is a corresponding elevation in carcass weight by 1.68 kg and 1.90 kg, respectively [53,54,55]. Significant progress has been achieved in China regarding the screening of genetic markers associated with vertebral traits in sheep [56,57,58,59,60,61,62]. For the sake of clarity, we compiled a concise summary of genes related to vertebral count and bone development, presented in Table 3.

## 4. Comparative Analysis of Overlapping Genes Linked to Vertebral Traits in Pigs, Donkeys, and Sheep

For detail discussion, we selected some overlapping genes, including NR6A1, VRTN, LTBP2, BMPs, and Hox, in donkeys, sheep, and pigs for their association with vertebral traits.

### 4.1. Nuclear Receptor Subfamily 6, Group A, Member 1 (NR6A1)

NR6A1, a member of the nuclear receptor family, exerts a pivotal role in regulating various biological processes such as growth, metabolism, and embryonic stem cell differentiation [65,66]. Furthermore, it is essential for orchestrating Hox signatures and determining the fate between neural and mesodermal cells within axial progenitors, which is crucial for vertebral column development in mice [65]. The involvement of NR6A1 in vertebral development extends beyond mice, as it has been extensively investigated in livestock species.

Studies in sheep [56,67], pigs [16,68,69], and donkeys [18] have consistently demonstrated the association between *NR6A1* and the number of vertebrae. Specific polymorphisms in the *NR6A1* gene, including IVS8-281G  >  A in intron 8 and rs414302710: A  > C in exon 8, have been identified as contributors to the variation in lumbar spine number and the number of lumbar vertebrae, particularly in Xinjiang Kazakh sheep [56,67]. These variations in vertebrae have significant implications for the carcass length and weight of Kazakh sheep [54]. Furthermore, in Dezhou donkeys, specific single-nucleotide polymorphisms (SNPs) within *NR6A1* have been linked to thoracic and lumbar vertebrae, highlighting the role of *NR6A1* in controlling the vertebral structure in this species [18]. Additionally, Fang et al. [70] reported a correlation between higher *NR6A1* gene expression and body size as well as carcass weight in Dezhou donkeys.

Pigs have also been a subject of investigation regarding the genetic basis of vertebral number, with *NR6A1* identified alongside *PLAG1* and *LCORL* as genes associated with this trait [69]. These findings have been further corroborated in European commercial pig breeds [71]. Moreover, a specific mutation, c.575T > C-*NR6A1*, has been linked to increased vertebral number in Licha Black and Laiwu pigs [72]. In Duroc × Landrace/Large White cross pigs, polymorphisms 748 C > T-NR6A1 and insertion g.20311_20312ins291-VRTN have been found to influence vertebral number [16], with similar associations verified in Chinese pigs [73]. Additionally, Zhang et al. [44] documented a positive correlation between NR6A1 and lumbar vertebrae in Large White × Minzhu pigs. These cumulative findings underscore the significance of NR6A1 in the regulation of vertebral development and its potential impact on livestock characteristics.

### 4.2. Vertnin (VRTN)

The significant association of the VRTN gene with the number of vertebrae and ribs has been extensively investigated in pigs. Consistently, Jiang et al. [13] systematically investigated the relationship between the g.20311_20312ins291 polymorphism within the VRTN gene and the augmentation of rib count. Their study revealed a consistent association between this polymorphism and an increase in the number of ribs. Additionally, Jiang et al. reported a positive correlation between rib count and carcass length (CL), body size, and cannon bone circumference. It is worth noting that cannon bone circumference is well-established for its role in enhancing body size traits, including the ability to support excessive body weight, engage in strenuous physical activity, and exhibit resistance to injuries. Multiple studies have reported this association [31,74,75,76], with consistent findings. Similarly, Xie L et al. [31,32] identified key candidate genes, including *HMGA1*, *VRTN*, and *BMP2*, linked to body length and size in pigs. They observed a positive correlation of *HMGA1* with leg bone size, *VRTN* with the number of vertebrae, and *BMP2* with the length of vertebrae. In line with these findings, other studies have also confirmed the association of the *VRTN* gene with skeletal development in pigs. Danish pigs were found to exhibit this association [75], as well as Duroc pigs [77], with an increase in the number of thoracic vertebrae and ribs. Consequently, Nakano et al. [78] reported that *VRTN* is not only associated with the number of thoracic vertebrae and length but also with carcass weight and body length in Duroc pigs. Furthermore, the pleiotropic effect of the *VRTN* gene has been documented, as it is associated with increased teat numbers and vertebral numbers in Chinese indigenous pigs [79,80,81]. Variations such as g.20311_20312ins291-*VRTN* have been linked to the number of ribs in Chinese Suhuai pigs [13] and the number of vertebrae in Duroc × Landrace/Large White crossbreeds [16]. Similar associations were found in Chinese Erhualian and White Duroc pigs [82] and in Duroc, Landrace, and Large White pigs [42]. Additionally, polymorphisms in *VRTN*-g.19034 A > C, *LTBP2* c.4481A > C, and *BMPR1A* genes have been linked to the number of thoracic vertebrae in Large White × Minzhu pig crossbreeds [40]. Zheng Y et al. [40] also found an association between SINE retrotransposon insertion polymorphism (sRTIP) in *VRTN* and the number of vertebrae [40].

In sheep, *VRTN* gene studies have also explored its association with the number of thoracic vertebrae [54,61]. These variations in vertebrae have been suggested to contribute to carcass and body length in sheep [54]. Notably, the polymorphism rs426367238-VRTN has been statistically correlated with thoracic vertebral numbers in Kazakh sheep [55]. In summary, the extensive body of research discussed here establishes a robust and consistent link between the *VRTN* gene and vertebral traits in both pigs and sheep. These findings contribute to our understanding of the genetic factors influencing skeletal development and provide valuable insights for breeding and selection programs in these livestock species.

### 4.3. Latent TGFβ Binding Protein-2 (LTBP2)

LTBP2, a pivotal component in the microfibril structure, exerts its influence on bone formation by virtue of its binding affinity with fibrillin 1 [83,84]. This interaction assumes critical significance as it actively regulates bone development through the intricate modulation of endogenous signaling pathways, particularly those governed by *transforming growth factor-β (TGF-β)* and *bone morphogenetic protein (BMP)* [85,86]. A seminal investigation by Park et al. [87] underscored the pivotal role played by LTBP2 in bone metabolism and its intriguing association with the number of thoracic vertebrae observed in porcine species [87].

Recent genetic inquiries have unraveled intriguing variations within the *NR6A1* and *LTBP2* genes that appear to account for the observed disparities in vertebral numbers between Xiang pigs and European pig breeds [34]. Furthermore, the involvement of *LTBP2* in shaping rib development has surfaced in multiple species, including sheep and pigs [60,76]. In a striking alignment with these findings, previous studies have posited *LTBP2* as an indirect regulator of *growth differentiation factor (Gdf11)*, a factor known to influence rib count in knockout mice [88]. Expanding the scope of *LTBP2*’s impact, a genome-wide association analysis in donkeys illuminated its potential involvement in determining vertebral numbers, with particular reference to Dezhou donkeys [22].

Further unraveling the genetic intricacies, Liu Z et al. [18] identified significant associations between specific *LTBP2* polymorphisms (c.5547 + 860 C > T, c.5251 + 281 A > C, c.3769 + 40 C > T, c.2782 + 3975 A > G) and thoracic vertebrae number. Additionally, variants at the c.1381 + 768 T > G and c.1381 + 763 G > T loci within LTBP2 have been correlated with lumbar vertebrae numbers [46]. These insights collectively contribute to our understanding of LTBP2’s multifaceted role in vertebral development and underscore its significance in shaping skeletal phenotypes across various species.

### 4.4. Bone Morphogenetic Proteins (BMPs)

**Figure 2:**
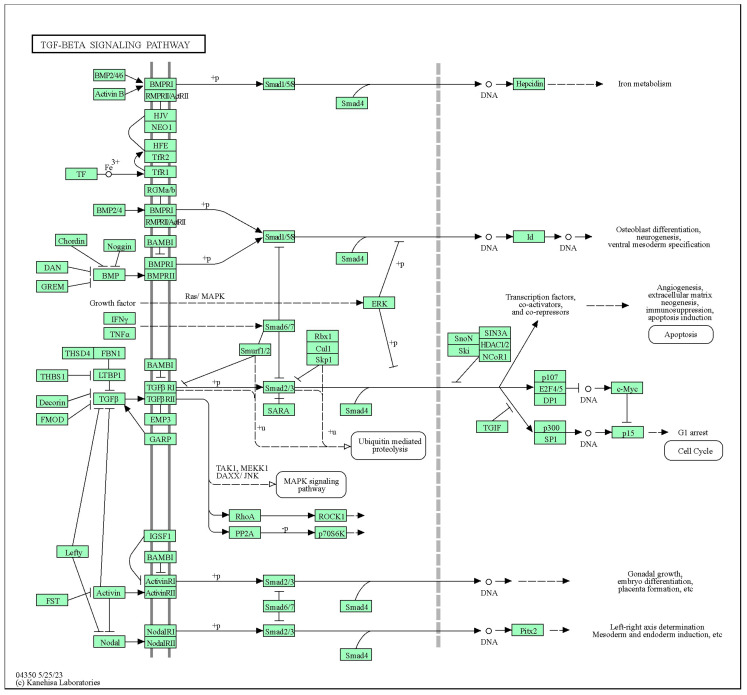
Regulatory mechanism of TGF-beta signaling pathway (hsa04350) in bone development.

*BMPs* belong to the transforming *growth factor (TGF)-β* superfamily of signaling molecules, known for their pivotal involvement in a wide spectrum of biological processes spanning from early embryonic tissue development to the maintenance of postnatal tissue equilibrium [89,90]. The principal mechanism through which *BMPs* elicit cellular responses is via the canonical signaling pathway, wherein intracellular Smads assume a central role in relaying extracellular signals to the cellular nucleus [91]. Notably, while *BMPs* engage the same Smads across diverse cell types, the functional diversity of BMPs is, in part, attributed to distinct transcription factors [92]. These transcription factors are recruited by Smads to orchestrate the regulation of specific subsets of target genes, a process contingent upon the cellular context, as shown in Figure 2 [93]. Within this group of transcriptional regulators, Hox proteins emerge as noteworthy constituents. Empirical investigations involving gain-of-function and loss-of-function experiments, coupled with the scrutiny of naturally occurring *Hox* gene mutations, have provided compelling evidence underscoring their indispensable contributions to embryonic skeletal patterning [94,95,96,97].

Numerous studies have consistently identified the significant association of a single nucleotide polymorphism (SNP), specifically rs320706814, within the *BMP2* gene, with its involvement in the regulation of growth and bone development [38]. Additionally, MBP4 has been correlated with an increase in carcass length of up to 4 cm in Duroc × (Landrace × Yorkshire) pig breeds [37], while *BMP15* was found to be associated with litter size in sheep [98]. This effect has also been observed in Duroc × (Landrace × Yorkshire) crossbreeds. Furthermore, a separate investigation reported that this SNP influences the length of vertebral structures and the size and development of hind leg bones in pigs [31,32].

The BMP receptors constitute a family of transmembrane serine/threonine kinases, which encompass the type I receptors *BMPR1A* and *BMPR1B.* These receptors serve as docking points for ligands belonging to the transforming *growth factor-beta (TGF-beta)* superfamily. Consistent with these findings, a GWAS study revealed that mutations in *BMPR1A* are associated with the number of thoracic vertebrae in Large White × Minzhu pig crossbreeds [39]. Recent GWAS studies have also shed light on the regulatory roles of *BMP7* and *BMP4* in bone development. These studies have elucidated their involvement in key signaling pathways, including in hippos (accession number: ssc04390), TGF-beta signaling pathways (accession number: hsa04350) and Wnt signaling pathways (accession number: hsa04310) in donkeys [10], and in mice [99,100,101,102]. Additionally, the association of BMP4 with limb and skeleton development, as well as tail formation, has been identified in Ethiopian fat-tailed sheep through the utilization of GWAS studies [64].

### 4.5. Homeobox (Hox) Genes

Hox genes have been recognized for their pivotal role in the determination of body segment identity throughout the course of embryonic development [103,104,105]. Furthermore, it is well established that *HOX* genes actively participate in shaping the anterior–posterior axis and contribute significantly to the intricate process of forming various anatomical structures, including limbs and the vertebral column [106,107]. Furthermore, studies involving gene mutation have demonstrated that variations in the sequence of the mouse *HOXC8* gene are linked to the addition of a thoracic vertebrae and an extra pair of ribs [108]. Similarly, differential expression of the *HOXC8* gene has been found to cause vertebral changes in livestock, resulting in the appearance of multi-vertebral variants in pigs [109] and Mongolian sheep [110]. Another investigation highlighted the significant role of *HOXB13* in the embryonic development of tendons, limbs, the skeleton, and tail formation in Ethiopian indigenous sheep [64]. Additionally, research has indicated a positive association between *HoxA* and the development of thoracic and lumbar vertebrae in Kazak sheep [61]. Recent studies have reported significant associations between polymorphisms in *HOX8* genes and body size, lumbar vertebrae length, and numbers in Dezhou donkeys [28]. Similarly, in pigs, the *HOX* family genes, including *HOXA10* and *HOXB7*, have been found to be associated with variations in thoraco-lumbar vertebrae [35,37]. Collectively, these findings highlight the critical role of *HOX* genes in vertebral development across various species and provide valuable insights into the molecular mechanisms governing this process.

## 5. Future Directions and Limitations

Based on the available published studies, our review article presented significant strides in exploring key genes associated with vertebral traits in pigs, donkeys, and sheep. The exploration of these genetic factors has opened up exciting avenues for further research and applications in animal breeding and genetics. Based on the facts and findings, one of the promising directions for future research in this domain is conducting more extensive genome-wide association studies (GWASs). GWASs have proven to be a valuable tool in identifying genetic variants linked to vertebral characteristics. These studies can help uncover additional variants contributing to the complexity of vertebral traits in various livestock species. The knowledge gained from such studies would provide a deeper understanding of the genetic intricacies governing vertebral development. Furthermore, it is crucial to advance our understanding of the functional characterization of the identified genes. This entails gene expression studies, knockout experiments, and pathway analysis to elucidate how these genes precisely influence vertebral development. Investigating the molecular mechanisms behind these genetic influences is pivotal for a comprehensive understanding of vertebral traits. Consequently, the genetic markers unearthed through these studies can be seamlessly integrated into selective breeding programs for livestock improvement. Breeders can employ this genetic information to make well-informed decisions about mating pairs with the aim of enhancing desirable vertebral traits in their herds or flocks.

Another intriguing avenue of research lies in comparative genomics across different livestock species. This approach can provide valuable insights into the evolutionary history of vertebral traits. It offers the opportunity to investigate why certain genes are more prominent in specific species and how these genetic factors have evolved to shape vertebral characteristics.

Finally, validating the effects of specific genetic variants on vertebral traits is essential. Modern genetic editing techniques, such as CRISPR/Cas9, offer a means to manipulate genes of interest and observe resulting phenotypic changes in vertebral development.

While these recent advancements are commendable, it is important to acknowledge the limitations and areas for further exploration. Presently, research primarily focuses on pigs, donkeys, and sheep. However, the diversity of livestock species with varying vertebral characteristics necessitates expanding the research to include species like horses and cattle. Furthermore, the genetic markers identified may not universally apply to all livestock breeds, and further research is imperative to encompass a broader spectrum of breeds and species. It is important to note that while genetics play a significant role, vertebral traits are polygenic and influenced by intricate environmental factors. Future research should consider the interplay between genes and the environment in shaping these traits. Donkeys remain relatively less explored in this context, emphasizing the need for comprehensive genetic investigations to fully comprehend vertebral traits in this species.

## 6. Conclusions

The research on genetic markers associated with vertebral traits in pigs, donkeys, and sheep has provided critical insights into the genetic basis of the number and length of vertebrae. Genes such as *PRKG2*, *NR6A1*, *LTBP2*, *VRTN*, *BMP*, and the *HOX* family genes have been identified as key players in controlling vertebral number and length, with specific genetic variants associated with these traits in donkeys, sheep, and pigs. This information offers a foundation for selective breeding strategies in these livestock species with the potential to enhance carcass quality and quantity. Moreover, the study of these genes highlights their pleiotropic effects and their significance in skeletal development across different organisms. Further research into the molecular pathways and interactions involved in vertebral traits is warranted to fully harness the potential of these genetic markers in improving livestock breeding programs.

## Figures and Tables

**Table 1:** Summary of genes associated with vertebral traits in pigs.

Genes	Associated Traits	Breeds	Country	Reference
*RSAD2-CMPK2*, *COL3A1*	✧ Bone and skeletal development	Meishan pigs	China	[30]
*HMGA1*, *VRTN*, *BMP2*	✧ Effects the number and length of vertebrae and the size of hind leg bones	Duroc × Landrace × Yorkshire crossbred pigs		[31,32]
*TIMP2*, *EML1*, *SMN1*	✧ Rib weights	Pigs		[33]
*NR6A1*, *LTBP2*	✧ Number of vertebrae	Xiang pigs		[34]
*GREB1L*, *ABCD4*, *VRTN*, *MIB1*	✧ Number of ribs	Beijing Black pigs		[27]
ABCD4 *Hox* family genes (*HOXB* 1–7, 9, and 13), *NTRK2*	✧ Vertebral number	Beijing Black pigs		[35]
*NR6A1*, *VRTN* *PLAG1*, *BMP2* *MC4R*	✧ Number of thoracic and lumbar vertebrae	Shanxia Black pigs		[36]
*HOXA10*	✧ Development of thoracolumbar vertebrae and rib primordium	Pigs		[37]
*BMP2*	✧ Growth and bone development ✧ Carcass length	Duroc × (Landrace × Yorkshire) hybrid pigs		[38]
*VRTN*, *LTBP2*, *BMPR1A*, *FOS*	✧ Number of thoracic vertebrae	White × Minzhu crossbred pigs		[39]
*VRTN*	✧ Number of vertebrae	Sujiang, Meishan, Bama, Erhualian, and Tibetan pigs		[40]
*VRTN*	✧ Number of ribs	Suhuai pigs		[13]
*VRTN*	✧ Number of thoracic vertebrae	Pigs		[41]
*VRTN*	✧ Number of vertebrae and ribs	Duroc, Landrace, and Large White pigs	Norway	[42]
*MMP9*, *VEGF*	✧ Pig femur and vertebra	Pigs	USA	[43]
*NR6A1*	✧ Lumber vertebrae	Large White × Minzhu pigs	China	[44]
*VRTN*, *FOS*, *PROX2*, *TGFB3*	✧ Number of thoracic vertebrae			

**Table 2:** Summary of genes associated with vertebral traits in donkeys.

Genetic Markers	Biological Effect	Breed	Country	Reference
*DCAF7*	✧ Number of thoracolumbar vertebrae	Dezhou donkeys	China	[11]
*PRKG2*	✧ Number of thoracic vertebrae ✧ The number and the length of lumbar vertebrae			[12]
*NR6A1*	✧ Number of lumber vertebrae			[18]
*LTBP2*	✧ Number of thoracic and lumbar vertebrae			[46]
*HOXC8*	✧ Number and length of lumbar vertebrae			[28]
*NLGN1*, *DCC*, *FBXO4 SLC26A7*, *TOX*, *LRP5* *WNT7A*, *LOC123286078*, *LOC123280142*, *GABBR2*, *LOC123277146*, *LOC123277359*, *BMP7*, *B3GAT1*, *EML2*	✧ Involved in Wnt and TGF-β signaling pathway regulation, which is linked with embryonic development or bone formation ✧ Number of thoracic vertebrae ✧ Number of lumbar vertebrae			[10]

**Table 3:** Summary of genes associated with vertebral traits and bone development in sheep.

Genes	Associated Traits	Breeds	Country	Reference
*NR6A1*	✧ Variation in the number of lumbar spine segments	Xinjiang Kazakh sheep	China	[56]
*SFRP4*	✧ It plays a role in bone development and is associated with the presence of multiple lumbar vertebrae	Duolang sheep		[57]
*SYNDIG1L*, *UNC13C*	✧ Influence the number of thoracic vertebrae	Han sheep and Sunite sheep		[52]
*TBXT*	✧ Associated with the determination of caudal vertebrae number and tail length	Sheep		[58]
*MGAT4A*, *KCNH1* *CPOX*, *CPQ*	✧ Play a role in determining the number of ribs in vertebrates	Hu sheep		[59]
*LTBP2*, *SYNDIG1L*	✧ Associated with the number of both ribs and vertebrae in vertebrates	Large fat-tailed sheep, Altay sheep, Tibetan sheep		[60]
*VRTN*, *HoxA*	✧ Linked with vertebral development and associated with the presence of thoracic vertebrae. They also play a role in regulating spinal development and morphology.	Xinjiang Kazakh sheep		[61]
*NDRG2*	✧ Associated with the development of the spine and provides valuable genetic resources for studying the transcriptome of various vertebral traits	Kazakh sheep		[62]
*VRTN*	✧ Correlated with thoracic vertebral number, carcass length, and carcass weight in vertebrates	China Kazakh sheep		[55]
*NID2*, *ACAN*	✧ Involved in skeletal development and the maintenance of cartilage structure	Afghani sheep	Iran	[63]
*ALX4*, *HOXB13*, *BMP4* *EYA2*, *SULF2*	✧ Involved in the embryonic development of tendons, bones, and cartilage as well as the development of limbs, the skeleton, and tail formation	Ethiopian indigenous sheep	Ethiopia	[64]

## Data Availability

All the data are available in the manuscript.
